# Programming of central and peripheral insulin resistance by low birthweight and postnatal catch-up growth in male mice

**DOI:** 10.1007/s00125-018-4694-z

**Published:** 2018-07-24

**Authors:** Lindsey M. Berends, Laura Dearden, Yi Chun L. Tung, Peter Voshol, Denise S. Fernandez-Twinn, Susan E. Ozanne

**Affiliations:** University of Cambridge Metabolic Research Laboratories and MRC Metabolic Diseases Unit, Wellcome Trust–MRC Institute of Metabolic Science, Level 4, Box 289, Addenbrooke’s Treatment Centre, Addenbrooke’s Hospital, Cambridge, CB2 0QQ UK

**Keywords:** Catch-up growth, Developmental programming, Diabetes, Hypothalamus, Insulin resistance, IUGR

## Abstract

**Aims:**

Intra-uterine growth restriction (IUGR) followed by accelerated postnatal growth is associated with an increased risk of obesity and type 2 diabetes. We aimed to determine central and peripheral insulin sensitivity in mice that underwent IUGR followed by postnatal catch-up growth and investigate potential molecular mechanisms underpinning their physiology.

**Methods:**

We used a C57BL/6J mouse model of maternal diet-induced IUGR (maternal diet, 8% protein) followed by cross-fostering to a normal nutrition dam (maternal diet, 20% protein) and litter size manipulation to cause accelerated postnatal catch-up growth. We performed intracerebroventricular insulin injection and hyperinsulinaemic–euglycaemic clamp studies to examine the effect of this early nutritional manipulation on central and peripheral insulin resistance. Furthermore, we performed quantitative real-time PCR and western blotting to examine the expression of key insulin-signalling components in discrete regions of the hypothalamus.

**Results:**

IUGR followed by accelerated postnatal growth caused impaired glucose tolerance and peripheral insulin resistance. In addition, these ‘recuperated’ animals were resistant to the anorectic effects of central insulin administration. This central insulin resistance was associated with reduced protein levels of the p110β subunit of phosphoinositide 3-kinase (PI3K) and increased serine phosphorylation of IRS-1 in the arcuate nucleus (ARC) of the hypothalamus. Expression of the gene encoding protein tyrosine phosphatase 1B (PTP1B; *Ptpn1*) was also increased specifically in this region of the hypothalamus.

**Conclusions/interpretation:**

Mice that undergo IUGR followed by catch-up growth display peripheral and central insulin resistance in adulthood. Recuperated offspring show changes in expression/phosphorylation of components of the insulin signalling pathway in the ARC. These defects may contribute to the resistance to the anorectic effects of central insulin, as well as the impaired glucose homeostasis seen in these animals.

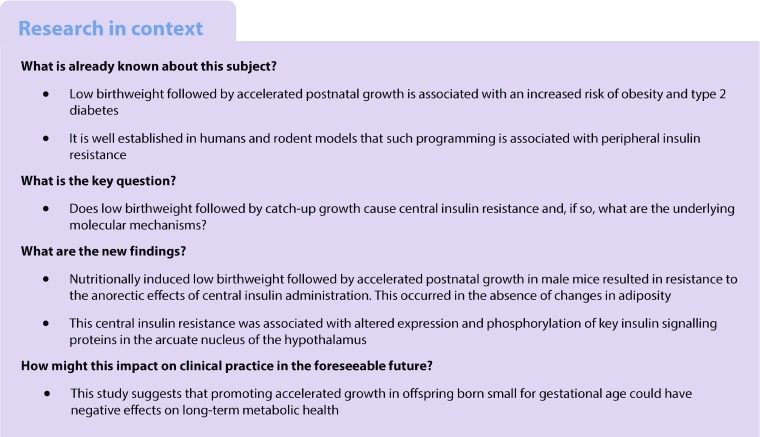



## Introduction

Obesity and type 2 diabetes are increasing globally at an unprecedented rate across all ages and sexes. Although a genetic basis for obesity susceptibility is undisputed, only a small proportion of the BMI variation within the population can be explained by known genetic variants, suggesting there is an interaction between genetic factors and the environment [1]. Evidence from clinical and experimental studies shows that the risk of developing many non-communicable diseases can be influenced by the early-life environment [2, 3]. Numerous studies have shown that low birthweight is associated with increased risk of developing impaired glucose tolerance and, subsequently, type 2 diabetes [4, 5]. Furthermore, it is well established that the accelerated growth that often follows low birthweight, as well as accelerated postnatal growth alone, are important risk factors for type 2 diabetes and obesity [6, 7]. Indeed, the combination of low birthweight with rapid weight gain, particularly if the baby crosses growth percentiles, is strongly linked to developing type 2 diabetes and obesity later in life [8, 9].

Insulin signalling is essential for maintaining whole-body energy homeostasis. The main sites of insulin action in the periphery are muscle and adipose tissue, where insulin increases glucose uptake; and liver, where insulin decreases glucose production. As recognised more recently, insulin signalling in the central nervous system is essential for maintaining energy homeostasis [10]. The hypothalamus is the primary site of insulin action in the brain [11]. In particular, agouti-related peptide/neuropeptide Y and pro-opiomelanocortin (POMC) neurons in the arcuate nucleus (ARC), and steroidogenic factor 1 (SF1) neurons in the ventromedial nucleus (VMH), have been shown to be important for hypothalamic insulin signalling [11, 12]. Neurons in the paraventricular (PVH) nucleus are also capable of sensing insulin [13], although the relevance of these neurons in maintaining glucose homeostasis is unknown.

Infants born small for gestational age (SGA) display defective insulin signalling in peripheral tissues, which may contribute to their increased type 2 diabetes risk [14, 15]. We have previously reported defects in the insulin signalling pathway in peripheral tissues of rodents that underwent IUGR followed by catch-up growth [16, 17] and similar differences in adipose and muscle biopsies from low-birthweight humans [14, 18]. Disrupted central insulin signalling, particularly in the ARC, causes defects in energy homeostasis, including disrupted glucose homeostasis and obesity [19]. Therefore, altered central insulin signalling may underlie the phenotypes reported in individuals that experience IUGR followed by catch-up growth. However, there are no studies that have yet addressed whether low birthweight followed by accelerated postnatal growth leads to central insulin resistance. This is not feasible to address in humans but can be analysed using animal models, which may also be used to determine whether relationships are causal.

Therefore, the aim of the current study was to examine whether murine offspring subjected to IUGR followed by postnatal catch-up growth show altered central insulin sensitivity, and to investigate the underlying molecular pathways mediating any programmed effects.

## Methods

### Animals

This research has been regulated under the Animals (Scientific Procedures) Act 1986 Amendment Regulations 2012 following ethical review by the University of Cambridge Animal Welfare and Ethical Review Body. C57BL/6J mice were purchased from Charles River (Harlow, UK). Female mice were mated at 6 weeks old. Dams were randomised to either a control diet (20% protein) or an isoenergetic low-protein diet (8% protein) fed ad libitum during gestation and lactation. Diet assignment was performed by an animal technician who was not involved in the outcome assessments.

Control and low-protein diets were from Arie Blok (Woerden, the Netherlands) [16]. Cross-fostering was performed at 3 days of age to establish two groups: control (offspring born to and suckled by dams fed a control-diet) and recuperated (offspring of low-protein-diet-fed dams nursed by control-diet fed dams). To maximise the effects of maternal nutritional differences on offspring postnatal growth (and recapitulate low birthweight followed by accelerated postnatal growth observed in humans), recuperated litters were culled to four pups and control litters were culled to eight pups on day 3. Body weights of animals were recorded at 3, 7, 14 and 21 days (control *n* = 30; recuperated *n* = 26) and weekly, thereafter (control *n* = 11; recuperated *n* = 8). Animals were caged in groups of two littermates and maintained on a 12 h light–dark cycle at 21–22°C with ad libitum access to chow diet (LAD1; Special Diet Services, Crawley, UK) and water. Ad libitum food intake in the home cage was measured weekly by calculating food consumed from food hoppers and the measurements were used to calculate cumulative food intake per mouse from 4–10 weeks of age. Because of the known effects of female oestrogen levels on insulin sensitivity [20] and the technical difficulties of oestrous cycle staging in mice, only male offspring were studied. All assessments were conducted at 12 weeks ± 3 days of age. For both groups, one male mouse in each litter underwent a different experimental procedure: (1) hyperinsulinaemic–euglycaemic clamp and related plasma analyses (control, *n* = 9; recuperated, *n* = 8); (2) body composition analysis and plasma and tissue collection (control, *n* = 11; recuperated, *n* = 8); (3) intracerebroventricular (ICV) insulin injection (control, *n* = 7; recuperated, *n* = 5); (4) GTT (control, *n* = 11; recuperated, *n* = 6).

### GTT

Mice were fasted for 16 h overnight and injected i.p. with a 10% (wt/vol.) glucose solution at 1 g/kg body weight. Blood glucose levels were measured from the tail vein using a hand-held glucose monitor before and 15, 30, 60, 120 and 180 min after glucose injection.

### Hyperinsulinaemic–euglycaemic clamp

After an overnight fast, animals were anaesthetised with an i.p. injection (10 μl/g) of Vetranquil (Novartis, Camberley, UK), Dormicum (Roche, Burgess Hill, UK), fentanyl (Martindale Pharma, Wooburn Green, UK) (VDF; 1:2:10 in 3 parts water) and the tail vein was cannulated. Programmable ‘Aladdin’ syringe pumps (World Precision Instruments, Hitchin, UK) were used for automated infusions of glucose and insulin via the tail vein. Blood glucose measurements and baseline blood samples were taken at t = −10 and t = 0 min via tail bleed. The clamp began with a primed-continuous (50 μl/h; 3.5 mU kg^−1^ min^−1^) i.v. infusion of insulin (Actrapid, Novo Nordisk, Gatwick, UK). During the 90 min insulin infusion, blood glucose was measured every 5 min for the first 20 min and every 10 min thereafter. To maintain euglycaemia relative to basal blood glucose levels, a variable i.v. infusion of 12.5% (wt/vol.) d-glucose was administered accordingly. The glucose infusion rate (GIR; μl/h) was used as an indicator of peripheral insulin action. Steady-state blood samples were taken at 10 min intervals during the final 30 min (t = 70 to t = 90 min) of the clamp, for determination of plasma glucose, insulin and NEFA.

### Plasma analysis

Blood samples were drawn from the tail tip into heparin-coated capillary tubes, chilled on ice and centrifuged to separate out plasma. Commercially available kits were used to determine plasma levels of insulin (Ultra-sensitive mouse insulin ELISA, Crystal Chem, IL, USA) and NEFA (Wako NEFA HR kit, Alphalabs, Eastleigh, UK). TNFα was measured by the MRC MDU Mouse Biochemistry Laboratory (Addenbrookes Hospital, Cambridge, UK). All measurements were carried out as technical duplicates and results were verified with a CV of <5%.

### Body composition analysis

Body composition was determined at 09:00 in fed animals using time-domain nuclear magnetic resonance (TDNMR; Minispec Plus, Bruker, Billerica, MA, USA). Body weight was recorded before TDNMR for calculation of relative fat mass and lean mass.

### Indirect calorimetry

Mice were placed in a Meta-Trace (Creative Scientific, UK) home cage measurement system linked to a Minimox system developed in house (P. Murgatroyd, Cambridge Institute of Metabolic Science and NIHR/Wellcome Trust Clinical Research Facility, Addenbrooke's Hospital, Cambridge, UK) to measure oxygen consumption (${\overset{\cdot }{V\mathrm{O}}}_2$) and carbon dioxide production ($\overset{\cdot }{V{\mathrm{CO}}_2}$). Ventilation rates were 390 ml/min and samples were collected every 18 min over 48 h. Energy expenditure (EE) was analysed over a 24 h period, using the equation: EE (J) = 15.818 × ${\overset{\cdot }{V\mathrm{O}}}_2$ + 5.176 × $\overset{\cdot }{V{\mathrm{CO}}_2}$ . The respiratory exchange ratio (RER) was calculated as $\overset{\cdot }{V{\mathrm{CO}}_2}$/${\overset{\cdot }{V\mathrm{O}}}_2$.

### Lateral ventricle cannulation

Mice were single-housed for 5 days prior to surgery and body weight recorded daily. Mice were anaesthetised with a mix of inhaled isoflurane and oxygen via nose cone and placed in a stereotactic device. A 26-gauge stainless-steel guide cannula (C315G-SPC; Plastics One, Roanoake, VA, USA) was implanted into the right lateral ventricle at +1.0 mm lateral, −0.5 mm caudal and −2.0 mm ventral to the bregma, fixed to the skull with cyanoacrylate adhesive and secured with dental cement (Associated Dental Products, Swindon, UK). To prevent occlusion, a dummy cannula (C315DC-SPC; Plastics One) was fitted. All animals received subcutaneous analgesia and antibiotic (Rimadyl, 5 mg/kg; Terramycin LA, 60 mg/kg; both Pfizer, Kent, UK). Body weight was monitored daily following surgery and intracerebroventricular insulin injections, with subsequent monitoring of food intake over a 20 h period, were performed 6–7 days later, once pre-surgical body weight had been recovered. Correct cannula placement was confirmed using an angiotensin-II thirst test (human A9525, Sigma-Aldrich, Gillingham, UK; 200 ng dose in saline [154 mmol/l NaCl] vehicle) immediately after the intracerebroventricular insulin injection experiment. Only animals that showed a rapid dipsogenic response (drink onset within 15 s to 3 min post injection) following angiotensin-II injection were included in the analyses.

### Intracerebroventricular insulin injection

Two hours before the onset of the dark period, food was removed from the cage and insulin (Actrapid; diluted in saline to 0.2 μU/μl) was injected into the lateral ventricle of awake, free-to-roam mice using a Hamilton syringe (Sigma-Aldrich) attached to the indwelling cannula. An insulin dose of 0.4 μU was selected based on trial data and previous reports of insulin-induced inhibition of food intake in mice [21, 22]. At the start of the dark period (18:00, t = 0), a pre-weighed quantity of food was returned to the cage and food intake was measured at t = 2, 4, 12 and 20 h following food presentation. For comparison, baseline food intake measurements were taken at the same time points the day prior to insulin injection; the dummy cannula was manipulated (removed and replaced) while mice were handled in the same way as for the insulin injection process, except no injection was performed. Pre-surgery food intake of single caged animals was calculated for the 12 h period immediately prior to surgery.

### RNA extraction, reverse transcription and quantitative PCR

Individual hypothalamic nuclei (ARC, PVH and VMH) were microdissected from 1 mm-thick coronal sections of frozen brains cut using a brain matrix (Braintree Scientific, Braintree, MA, USA). After microdissection, RNA was extracted using RNeasy MinElute columns with on-column DNase treatment according to the manufacturer’s instructions (Qiagen, Manchester, UK), and RNA was reverse transcribed using a High-Capacity cDNA Reverse Transcription kit (Thermo Fisher, Loughborough, UK). *Insr*, *Irs1*, *P85α* (also known as *Pik3r1*) and *Pik3cb* mRNA expression was measured in technical duplicates using SYBR quantitative PCR (Thermo Fisher). *Irs2* and *Ptpn1* (which encodes protein tyrosine phosphatase 1B [PTP1B]) mRNA was measured using TaqMan assay-on-demand (*Irs2*: Mm03038438_m1; *Ptpn1*: Mm00448427_m1; both Thermo Fisher). Target gene expression was normalised to the housekeeping gene *Sdha* (expression of which did not differ between groups). All quantitative PCR was performed using a StepOne Plus qPCR machine (Thermo Fisher).

### Protein extraction, SDS-PAGE and western blotting

ARC was microdissected as above. Protein was extracted in lysis buffer (50 mmol/l HEPES [pH 8], 150 mmol/l NaCl, 1% (wt/vol.) Triton X-100, 1 mmol/l Na_3_VO_4_, 30 mmol/l NaFl, 10 mmol/l Na_4_P_2_O_7_, 10 mmol/l EDTA (all Sigma-Aldrich) and a 1:200 dilution of protease inhibitor cocktail set III [Merck-Millipore, Watford, UK]). Total protein concentration of lysates was determined using a bicinchoninic acid kit (Sigma-Aldrich) and samples diluted in Laemmli buffer. Samples (4 μg protein) were loaded onto 8% polyacrylamide gels for electrophoresis and transferred to a polyvinylidene difluoride (PVDF) membrane (Merck-Millipore). The membrane was washed, then stained with Ponceau red (Sigma-Aldrich). The stained membrane was imaged and bands measured for normalisation. Membranes were blocked in 5% (wt/vol.) BSA in Tris buffered saline with Tween 20 (TBST), followed by overnight incubation with the following primary antibodies: insulin receptor β subunit (sc-711, Santa Cruz Biotechnology, Dallas, TX, USA; 1:200 dilution), p-IRS1 Ser307 (07-247, Upstate Biotechnology, Lake Placid, NY, USA; 1:1000 dilution) and phosphoinositide 3-kinase (PI3K) p110β (ab151549, Abcam, Cambridge, UK; 1:1000 dilution). All antibodies were validated as cross-reactive to mouse. Following 3 × 5 min washes in TBST, membranes were incubated for 1 h in horseradish-peroxidase (HRP)-linked secondary antibody (Abcam) diluted 1:10,000 in 5% BSA in TBST. Antibody binding was detected using chemiluminescence substrate and the ChemiDoc Imaging system (Bio-Rad, Hemel Hempstead, UK). Protein abundance was quantified by band densitometry using Image Lab 5.1 software (Bio-Rad) and normalised to total transferred protein as visualised by Ponceau stain.

### Statistical analysis

All data were analysed using Prism 7 software (GraphPad, La Jolla, CA, USA). Values are expressed as mean ± SEM. For data comparing one variable between offspring, an unpaired *t* test was used. In the ICV insulin injection experiments, a paired *t* test was used to compare measurements at baseline and after insulin injection from the same animals. For experiments measuring data over time, a two-way ANOVA with repeated measures was used to determine the overall effect of maternal diet and time. A Sidak post hoc test was carried out if the overall *p* value for two-way ANOVA was < 0.05.

## Results

### Growth trajectory of recuperated offspring

**Fig. 1:**
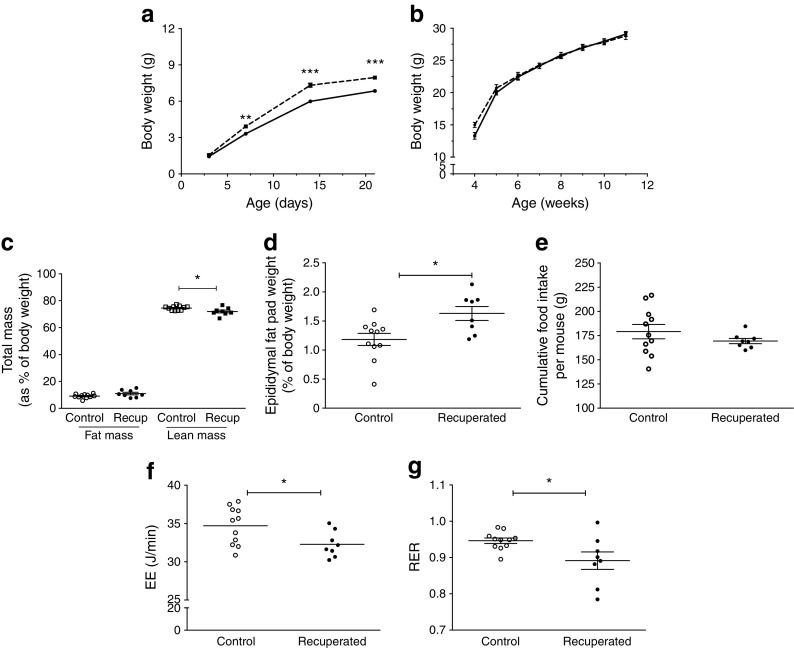
Postnatal growth trajectory of control and recuperated offspring. (**a**) Body weight, recorded on postnatal day 3, 7, 14 and 21. *p* < 0.001 for overall effect of maternal diet and for overall effect of time, two-way ANOVA. (**b**) Post-weaning body weight. (**c**) Fat mass (circles) and lean mass (squares) of 12-week-old offspring as measured by TDNMR, expressed as a percentage of total body weight. (**d**) Epididymal fat pad weight of 12-week-old offspring, expressed as percentage of total body weight. (**e**) Cumulative food intake of offspring from 4 to 10 weeks of age. (**f**) EE over 24 h in offspring at 12 weeks of age. (**g**) RER of offspring at 12 weeks of age. Solid line/white symbols, control; dashed line/black symbols, recuperated. In (**a**), control *n* = 30, recuperated *n* = 26; in (**b**–**g**), control *n* = 11, recuperated *n* = 8. In (**a**), **p < 0.01, ***p < 0.001, post hoc test, recuperated vs control; in (**c**, **d**, **f**, **g**), *p < 0.05, *t* test. Recup, recuperated

Recuperated offspring showed accelerated postnatal growth and were significantly heavier than controls by the end of the first postnatal week (Fig. 1a). Recuperated offspring remained significantly heavier than controls throughout the remainder of the lactation period (*p* < 0.001). However, there was no difference in body weight between control and recuperated offspring from 4 to 12 weeks of age, or in whole-body fat mass as measured by TDNMR (as a percentage of body weight) at 12 weeks of age (Fig. 1b,c). Despite no change in overall adiposity, recuperated offspring had a significantly larger epididymal fat depot than controls when expressed as a percentage of whole body weight (*p* < 0.05; Fig. 1d).

To assess energy balance, ad libitum cumulative food intake per mouse in the home cage was measured weekly for the period of 4–10 weeks of age. There was no difference in cumulative food intake between control and recuperated offspring (Fig. 1e). At 12 weeks, EE was lower in recuperated offspring compared with controls (*p* < 0.05; Fig. 1f). The RER was also significantly lower in recuperated offspring compared with controls (*p* < 0.05; Fig. 1g).

### Recuperated offspring show reduced glucose tolerance and insulin sensitivity

**Fig. 2:**
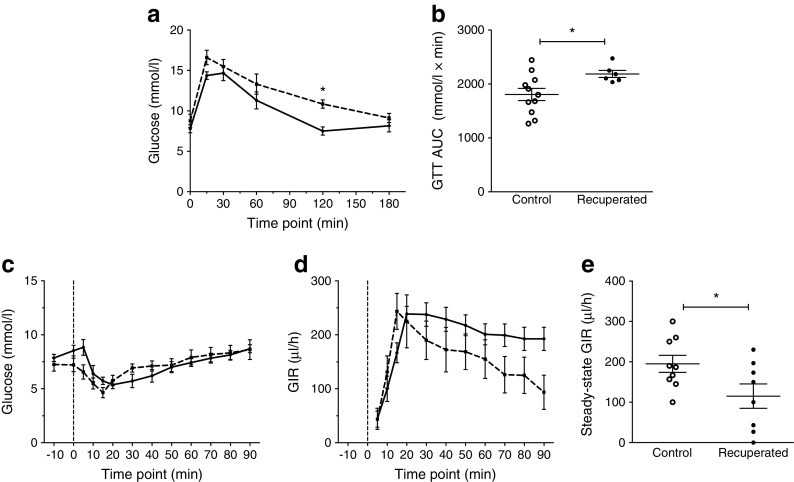
Glucose and insulin sensitivity of control and recuperated offspring. (**a**) GTT (1 g/kg i.p. glucose injection) in offspring at 12 weeks of age. *p* < 0.001 for overall effect of maternal diet and *p* < 0.01 for overall effect of time, two-way ANOVA. (**b**) AUC of GTT. (**c**) Plasma glucose levels during hyperinsulinaemic–euglycaemic clamp in offspring at 12 weeks of age. Insulin bolus represented by the vertical dashed line at time 0. (**d**) GIR during hyperinsulinaemic–euglycaemic clamp study in offspring at 12 weeks of age. Insulin bolus represented by the vertical dotted line at time 0. *p* < 0.01 for overall effect of maternal diet and *p* < 0.001 for overall effect of time, two-way ANOVA. (**e**) GIR during hyperinsulinaemic steady state. Solid line/white circles, control; dashed line/black circles, recuperated. In (**a**, **b**), control *n* = 11, recuperated *n* = 6; in (**c**–**e**), control *n* = 9, recuperated *n* = 8. In (**a**), *p < 0.05, post hoc test, recuperated vs control; in (**b**, **e**), *p < 0.05, *t* test

Control and recuperated offspring underwent an i.p. GTT at 12 weeks of age. There was an overall effect of maternal diet on glucose levels during the test (*p* < 0.001; Fig. 2a). Blood glucose was significantly higher in recuperated animals 120 min following i.p. glucose injection compared with controls (*p* < 0.05). Furthermore, recuperated offspring had a significantly higher AUC over the course of the GTT compared with controls (*p* < 0.05; Fig. 2b).

To determine whole-body insulin sensitivity, hyperinsulinaemic–euglycaemic clamps were carried out in offspring. Compared with baseline, in both groups, plasma insulin levels increased significantly following insulin infusion (control: 75.86 ± 6.90 to 901.70 ± 96.55 pmol/l; recuperated: 68.96 ± 10.34 to 665.50 ± 110.34 pmol/l; *p* < 0.01 compared with baselines). Plasma glucose levels were not significantly different at baseline and hyperinsulinaemic steady states in control or recuperated groups (Fig. 2c). There was a significant effect of maternal diet on GIR throughout the clamp (Fig. 2d), with GIR lower in recuperated offspring (*p* < 0.01). Furthermore, mean GIR during steady state was lower in recuperated offspring compared with controls (*p* < 0.05; Fig. 2e). Compared with baseline, plasma NEFA levels were significantly lower during hyperinsulinaemia in both groups (control: 0.44 ± 0.05 to 0.20 ± 0.04 mmol/l; recuperated: 0.27 ± 0.04 to 0.16 ± 0.04 mmol/l; *p* < 0.001 compared with baseline); the percentage drop in NEFA levels from baseline to hyperinsulinaemic steady state was not significantly different between groups (control: 56.2% ± 5.3%; recuperated: 42.8% ± 6.6%).

### Recuperated mice are resistant to the anorectic effects of central insulin

**Fig. 3:**
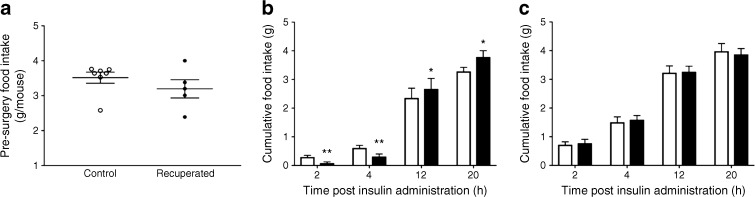
Effects of ICV insulin administration on food intake in control and recuperated offspring. (**a**) Pre-surgery food intake of control (white circles) and recuperated (black circles) offspring, measured for the 12 h period immediately before the ICV cannula was implanted. (**b–c**) Cumulative food intake was measured at 2, 4, 12 and 20 h after food presentation at baseline conditions (white bars) or after insulin injection (0.4 μU; black bars) into the lateral ventricle of control offspring (**b**) and recuperated offspring (**c**). Control *n* = 7; recuperated *n* = 5). *p < 0.05, **p < 0.01, *t* test vs baseline

There was no difference in pre-surgery baseline food intake over a 12 h period between control and recuperated offspring (Fig. 3a). ICV insulin injection resulted in a significant reduction in food intake in control mice during the first 4 h compared with baseline (*p* < 0.01; Fig. 3b). This was followed by hyperphagia compared with baseline food intake from 12 to 20 h after food presentation (*p* < 0.05). In contrast to control animals, there was no effect of insulin on food intake compared with baseline in recuperated offspring (Fig. 3c). There was no effect of insulin injection on body weight in response to ICV insulin injection in either group (data not shown).

### Expression of insulin signalling pathway components in hypothalamic nuclei

**Fig. 4:**
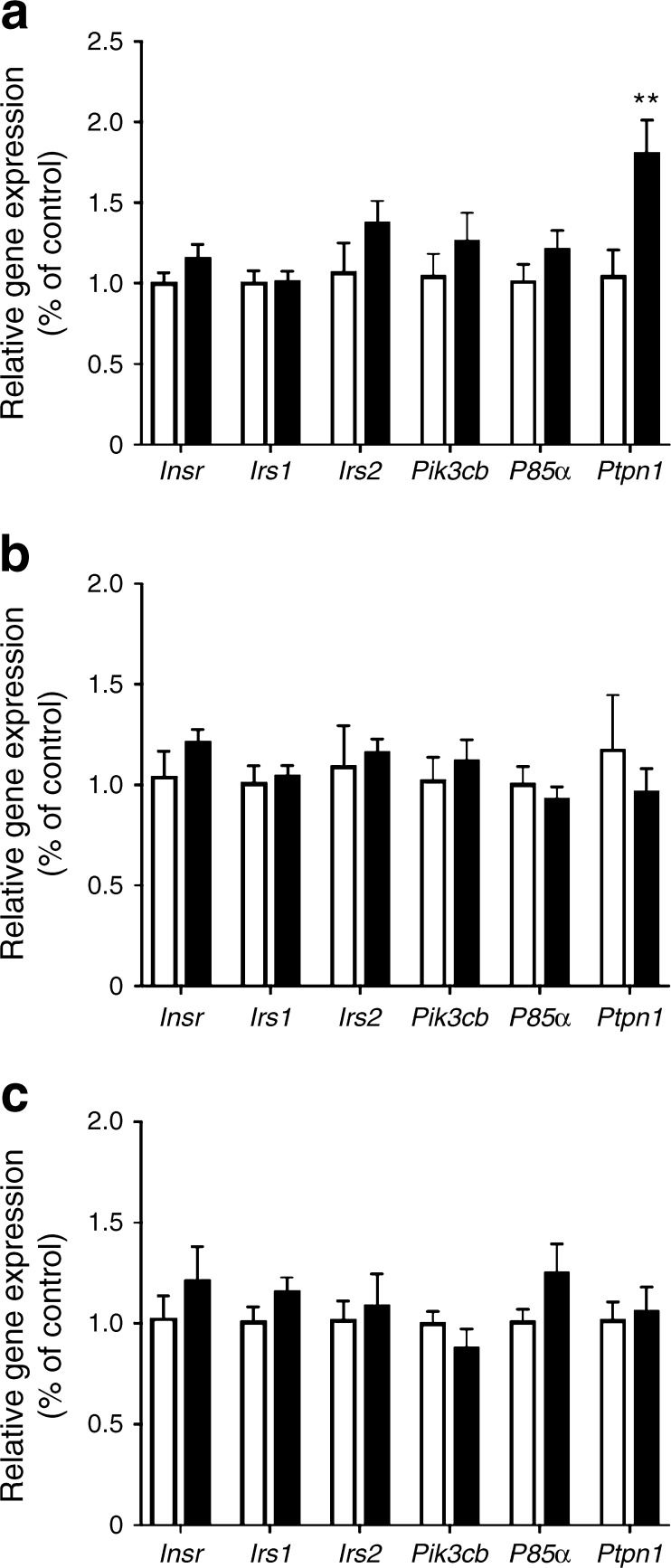
Insulin-signalling-related gene expression in the ARC, PVH and VMH of control and recuperated offspring. Expression of insulin-signalling-related genes encoding insulin receptor (*Insr),* IRS1 (*Irs1),* IRS2 *(Irs2),* PI3K catalytic subunit *(Pik3cb),* PI3K regulatory subunit *(P85α)* and PTP1B (*Ptpn1)* in the (**a**) ARC, (**b**) PVH and (**c**) VMH of 12-week-old control (white bars) and recuperated (black bars) offspring. mRNA levels were normalised to housekeeping gene *Sdha*, expressed as relative to control. Control *n* = 7; recuperated *n* = 8. **p < 0.01, *t* test vs control

Gene expression of components of the insulin signalling pathway were measured in distinct hypothalamic nuclei capable of sensing and integrating insulin signalling into an alteration of food intake. There was no difference in the expression of *Insr* (encoding the insulin receptor), *Irs1* (encoding IRS1) or *Irs2* (encoding IRS2) in the ARC, VMH or PVH between control and recuperated offspring (Fig. 4a–c). There was also no difference in the expression of *Pik3cb* and *P85α*, which encode the catalytic and regulatory subunits of PI3K, respectively, in any of the nuclei examined (Fig. 4a–c). We next examined the expression of PTP1B (encoded by *Ptpn1*), which is known to negatively regulate insulin signalling in the hypothalamus [19]. There was a significant increase in *Ptpn1* expression in the ARC (*p* < 0.01; Fig. 4a) but not PVH or VMH (Fig. 4b,c). Previous studies have shown that PTP1B expression is increased by TNFα, the levels of which are often associated with increased inflammation seen with obesity [23]. However, there was no difference in serum TNFα levels between control and recuperated offspring (control: 7.84 ± 0.52 ng/ml; recuperated: 9.06 ± 0.57 ng/ml) in the present study.

**Fig. 5:**
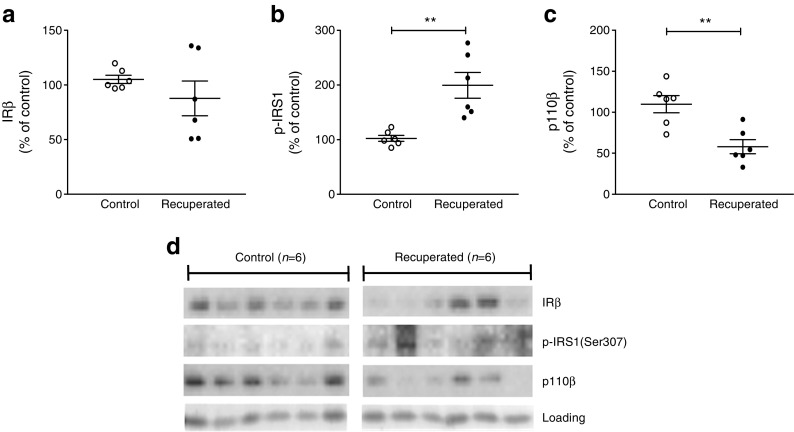
Levels of key insulin-signalling proteins in the ARC of control and recuperated offspring. Expression of insulin signalling pathway proteins (**a**) insulin receptor (IRβ), (**b**) phospho-IRS1 (Ser307) and (**c**) p110β catalytic subunit of PI3K in the ARC of 12-week-old control (white circles) and recuperated (black circles) offspring (relative protein levels normalised to whole protein loading [as assessed by Ponceau stain], expressed as percentage of control). (**d**) Western blot images of IRβ, p-IRS1 and p110β and loading controls. Control *n* = 6; recuperated *n* = 6. **p < 0.01, *t* test

There was no difference in the expression of the insulin receptor in the ARC in recuperated offspring (Fig. 5a). However, recuperated offspring showed increased levels of IRS1 phosphorylated at Ser307 (P-IRS1; *p* < 0.01; Fig. 5b) and decreased levels of the p110β catalytic subunit of PI3K (*p* < 0.01; Fig. 5c) in the ARC. Antibody staining for each protein, along with loading control, are shown in Fig. 5d.

## Discussion

We used a rodent model to show that animals that undergo IUGR followed by rapid postnatal catch-up growth display whole-body insulin resistance in adulthood. This is associated with peripheral insulin resistance, as demonstrated by the results of the hyperinsulinaemic–euglycaemic clamps. Our findings show, for the first time, that suboptimal nutrition during early life can also lead to central insulin resistance, and identify a novel mechanism in the ARC that may underpin this. Previous rodent models have shown that raising neonates in small litters disrupts energy homeostasis and causes insulin resistance in neuronal cultures [24]. Therefore, the phenotype in recuperated offspring could be due either to the low birthweight caused by IUGR or to the accelerated neonatal growth caused by the small litter rearing, or indeed a combination of the two (as in the human situation where most low birthweight babies will experience postnatal catch-up growth).

Recuperated offspring had higher glucose levels during the GTT, indicating decreased glucose tolerance, in agreement with other rodent and larger mammal models of nutritional programming [25–28]. This glucose intolerance is likely attributed to reduced insulin sensitivity, as these mice also required a lower GIR to maintain blood glucose during the hyperinsulinaemic–euglycaemic clamp. We have previously reported that recuperated offspring show molecular defects in expression of key components of the insulin signalling pathway in peripheral tissues [16, 17], thus this may contribute to the insulin resistance shown in the clamp. Despite no changes in total body weight or adiposity, recuperated offspring showed an increase in epididymal fat mass. This is in agreement with previous reports from human studies that infants born SGA have higher central adiposity, despite a comparable BMI to infants born at a normal weight [29]. Recuperated offspring demonstrated reduced EE, which may predispose them to an increase in body weight, as well as total adiposity, as they get older. Our data from indirect calorimetry also suggest different fuel usage in recuperated offspring as their RER was lower, indicating that fats are preferentially used as a substrate for energy generation compared with carbohydrates.

In this study, control mice were sensitive to the anorectic effects of ICV insulin; food intake was significantly reduced 2 and 4 h after food presentation when insulin was administered into the lateral ventricle. This is in agreement with several studies showing the anorectic effects of central insulin administration, either intranasally [30] or directly to the brain via injection [31–33]. However, there was no effect on food intake in recuperated mice, showing that these animals were resistant to the anorectic effects of exogenous insulin and suggesting central insulin resistance. Studies in both rodents and humans have shown that central insulin resistance contributes to the pathology of whole-body insulin resistance [10, 34]. Therefore, the central insulin resistance observed in recuperated offspring could contribute to the whole-body insulin resistance observed.

Although central insulin resistance is associated with obesity and type 2 diabetes [35, 36], it is usually thought of as a consequence of chronic hyperinsulinaemia and obesity rather than a cause. However, it has been shown in rodents fed a high-fat diet (HFD) that resistance to ICV insulin can occur within a matter of days and before an increase in adiposity [37]. In this previous study of the effects of an acute HFD, central insulin resistance was associated with elevated NEFA. NEFA are able to cross the blood–brain barrier and activate inflammatory pathways, resulting in hypothalamic insulin resistance [38]. Interestingly, in our study, recuperated offspring develop central insulin resistance despite being maintained on a regular chow diet and in the absence of elevated NEFA levels. The presence of insulin resistance in the absence of frank obesity, elevated NEFA or hyperinsulinaemia raises the possibility that changes to insulin sensitivity in these mice are programmed during early life. There is evidence from human studies that the fetal brain responds to changes in maternal glucose in insulin-sensitive but not insulin-resistant mothers, suggesting that fetuses of insulin-resistant mothers are themselves insulin resistant in utero [39]. Furthermore, babies from obese pregnancies display insulin resistance at birth [40]. Therefore, it is possible that in circumstances of altered metabolic state in the mother during pregnancy, insulin resistance programmed during the perinatal period is causative of further metabolic dysfunction, rather than being a consequence of it.

Our previous studies in adipose tissue and muscle from recuperated offspring [16, 17] and from humans with a low birthweight [14] have demonstrated changes in expression of key insulin signalling proteins. One of the biggest effects is observed on levels of the p110β catalytic subunit of PI3K. In humans and rodents, the reduced expression occurs at the post-transcriptional level, with no differences being observed at the mRNA level. Here, for the first time, we demonstrate that this protein is also present at substantially reduced levels in the ARC of recuperated animals. In addition to reduced p110β, we observed an increase in serine phosphorylation of IRS-1. *Ptpn1* mRNA levels were also increased in the ARC from recuperated animals. Whole-body or neuronal deletion of *Ptpn1* improves insulin sensitivity [19]. Therefore, all of these changes could contribute to the lack of anorectic response to ICV insulin in recuperated offspring. As intact insulin signalling in the ARC is essential for maintaining energy homeostasis, ARC insulin resistance also likely contributes strongly to the peripheral insulin resistance observed in the hyperinsulinaemic–euglycaemic clamps in recuperated offspring. A recent study has shown that insulin signals through POMC neurons to cause browning of white adipose tissue and therefore increased EE [41]. Abrogation of insulin signalling by overexpression of PTP1B in the ARC inhibits this process. Conversely, mice with a global knockout of *Ptpn1* have increased EE [42]. Therefore, the increased *Ptpn1* expression we observed in the ARC may also contribute to the decreased EE that we observed in recuperated offspring.

The specific increase in *Ptpn1* expression in the ARC but not in other hypothalamic areas examined suggests selective insulin resistance. Selective ARC resistance to leptin has been reported previously as a consequence of diet-induced obesity [43]. To our knowledge, there have not been reports of selective insulin resistance within the different nuclei of the hypothalamus. However, selective hypothalamic insulin resistance has been reported in humans, as obese men show responses in cognitive areas but not hypothalamic areas of the brain after intranasal insulin administration [44].

We have previously shown that, compared with control animals, recuperated animals have a reduced lifespan and show signs of accelerated cellular ageing [45]. Central insulin responsiveness is decreased with ageing [46], which may in part be due to increased negative regulation of insulin signalling transduction. The expression of PTP1B, as well as other phosphatases that negatively regulate insulin signalling, is increased with ageing and has been suggested as an underlying mechanism of age-associated insulin resistance [47–49]. The increase in *Ptpn1* expression in the ARC and central insulin resistance could therefore be further examples of accelerated ageing in mice that have undergone IUGR followed by catch-up growth.

In conclusion, our results show that in mice, undernutrition during the in utero period followed by rapid neonatal catch-up growth causes central insulin resistance and peripheral insulin resistance. This has important implications for the long-term health of an individual. A recent study has shown that humans with central insulin resistance have a reduced capacity to lose weight by lifestyle intervention [50]. Furthermore, it is unclear whether primary treatments for improving insulin sensitivity, such as thiazolidinediones and metformin, can cross the blood–brain barrier and improve brain insulin sensitivity. Therefore, humans with central insulin resistance have a reduced capacity for metabolic improvements via both lifestyle and pharmaceutical interventions. These findings in mice, if extrapolated to humans, suggest that individuals exposed to a suboptimal early environment may be less responsive to currently available interventions.

## Data Availability

All data generated or analysed during this study are included in this published article.

## Abbreviations

ARC: Arcuate nucleus
EE: Energy expenditure
GIR: Glucose infusion rate
HFD: High-fat diet
ICV: Intracerebroventricular
IUGR: Intra-uterine growth restriction
PI3K: Phosphoinositide 3-kinase
POMC: Pro-opiomelanocortin
PTP1B: Protein tyrosine phosphatase 1B
PVH: Paraventricular nucleus
RER: Respiratory exchange ratio
SGA: Small for gestational age
TBST: Tris buffered saline with Tween 20
TDNMR: Time-domain nuclear magnetic resonance
VMH: Ventromedial nucleus
